# Posttraumatic stress symptoms in families of cancer patients admitted to the intensive care unit: a longitudinal study

**DOI:** 10.1186/s40560-016-0162-3

**Published:** 2016-07-20

**Authors:** Miyuki H. Komachi, Kiyoko Kamibeppu

**Affiliations:** Division of Health Sciences, Graduate School of Health and Welfare Sciences, International University of Health and Welfare, 1-3-3 Minamiaoyama Aoyama 1-Chome Tower 4th and 5th floor, Minato-ku Tokyo, 107-0062 Japan; Department of Family Nursing, Division of Health Sciences and Nursing, Graduate School of Medicine, The University of Tokyo, 7-3-1 Hongo, Bunkyo-ku Tokyo, 113-0033 Japan

**Keywords:** Intensive care, Family, Posttraumatic stress disorder, Cancer, Recurrence

## Abstract

**Background:**

Families of cancer patients in the ICU often experience severe stress. Understanding their experience is important for providing family-centered care during this difficult period. Little is known about the experience of families of cancer patients admitted to the ICU. This study evaluated the prevalence of posttraumatic stress symptoms (PTSS) among families of cancer patients admitted to the ICU.

**Methods:**

We carried out a longitudinal study at a teaching and advanced treatment hospital. Participants were 23 family members of 23 ICU patients. Family members provided demographic data, electronic medical records of patients, and completed the Impact of Event Scale-Revised (IES-R), the Center for Epidemiologic Studies Depression Scale (CES-D), and the State-Trait Anxiety Inventory Form X (STAI-state, trait).

**Results:**

Mean total IES-R total score, IES-R re-experience score, IES-R avoidance score, and STAI-state score within 24 h of ICU admission and 3 months later differed significantly. The IES-R score of families of patients with recurrent cancer was significantly higher than the score of families of patients with an original cancer diagnosis (*t* = 2.63, *p* = 0.029). For two-way analysis of variance, time point was significantly associated with IES-R score (*F* = 1.751, *p* = 0.011, *df* = [1]).

**Conclusions:**

Families of recurrent cancer patients admitted to the ICU experience serious PTSS within 24 h of admission. It is important that appropriate psychiatric support be provided to family members of these patients.

## Background

When patients with cancer are admitted to the ICU after invasive surgical procedures, they receive high-quality, specialized medical treatment [1].

According to a survey of healthcare facilities and a bedside overview in Japan, the number of operations performed on patients with cancer increased from 2010 to 2014, and the number of ICU beds at the cancer institute hospital of Japanese Foundation for Cancer Research occupied by these patients increased from 2011 to 2014 [2, 3]. Results of an investigation in Japan revealed that 40 % of patients in the ICU died of cancer [4].

Patients admitted to the ICU may not be able to communicate for several reasons such as sedation, ventilator use, delirium, or coma. Families of patients in the ICU experience severe stress [5]. Recent studies show that family members experience severe mental stress during the initial period after a patient’s admission to the ICU in Brazil, Europe, and the USA [6–8].

Some studies reported that families of patients in the ICU with cancer in Japan have psychiatric stress [9]. Families of patients with recurrent cancer experienced more severe psychological shock than families of patients with original cancer at a general ward in Japan and Spain [10, 11]. The results of these studies may indicate that families of patients admitted to the ICU with recurrent cancer may experience a severe psychological burden. However, few studies have quantitatively analyzed the psychiatric stress of family members of cancer patients admitted to the ICU with recurrent disease.

It is also important to study posttraumatic stress of family members on a continuous basis, from early ICU admission until the patient leaves the ICU to determine if stress experienced at an early point is a predictive factor for posttraumatic stress after 3 months [12]. The aims of this study were (1) to investigate the prevalence of families with PTSS and associated symptoms within 24 h of ICU admission and after 3 months and (2) to examine the prevalence of families with PTSS among patients with an original diagnosis of cancer compared with patients with a diagnosis of recurrent cancer at the same time points.

## Methods

### Setting and participants

This longitudinal study was performed and consecutively recruited at a teaching and advanced treatment hospital. We conducted the survey and collected pertinent medical records in the medical/surgical ICU (23 beds).

Inclusion criteria for patients were ICU planned admission after original or recurrent cancer surgery. The only exclusion criterion for patients was living alone. Inclusion criteria for family members included (1) the family member visited the patient in the medical/surgical ICU within 24 h of admission; (2) the family member was the patient’s spouse, child, parent, sibling, or relative; (3) the family member granted permission to be surveyed by a physician and a ICU staff; (4) the family member was older than 20 years; (5) the family member was able to provide informed consent; and (6) the family member was able to communicate in Japanese. Exclusion criteria for family members included (1) being under treatment for a mental or physical disease and (2) being a caregiver for other family members.

We fully explained our research and asked for a list of participants who satisfied the inclusion criteria for patients and family members. We requested that participants return the study questionnaires sent by mail within 24 h (time point 1: T1) and 3 months later (time point 2: T2) to avoid placing pressure on them to complete the information in front of us and to protect the participants’ anonymity. Within 2 weeks of T2, a postcard with a written reminder about filling out the follow-up questionnaire for T2 was sent to participants. Thereafter, the questionnaire and pre-stamped/pre-addressed envelopes to encourage people to return the questionnaires for T2 were sent to participants. We asked participants to fill out questionnaires within 24 h of a family member’s admission to the ICU and 3 months later. Postmarks of all questionnaires analyzed were the day of or the day after the participant was recruited at T1 and T2.

### Data collection

To investigate the psychiatric conditions of family members quantitatively, we used data of subjects gathered from electronic medical records and self-reported questionnaires.

The questionnaires asked the following: demographics of the family (age, sex, number of live-in members, education level, marital status, family relationship to patient, and household income); the number of family members who had ever died in an ICU; personal history of mental disorder, other family members’ history of mental disorder, experience with loss of a family member, other experience with loss, occurrence of a recent stressful event, and history of traumatic stress. For this latter variable, we used an event checklist that was published in the Clinical-Administered posttraumatic stress disorder (PTSD) scale for DSM-IV [13].

The following were collected from electronic medical records: patient demographics (age and sex); clinical characteristics (APACHE II score) [14], length of ICU stay, reason for admission to ICU, number with complications, number who died during ICU stay, and history of ICU admissions.

In addition, participants were asked to complete the existing measures of the Japanese-Language Version of the Impact of Event Scale-Revised (IES-R-J, hereafter referred to as IES-R) [15, 16], the Center for Epidemiologic Studies Depression Scale (CES-D) [17, 18], and the Spielberger’s State-Trait Anxiety Inventory Form X (STAI-state, trait) [19, 20].

The IES-R was used to measure PTSS in the families of cancer patients admitted to the ICU [15, 16]. The IES-R has been tested in various studies, including the survey of the Great Hanshin-Awaji Earthquake, and is accepted as a reliable and valid scale for measurement of symptoms related to PTSD among Japanese subjects [15]. The IES-R items comprised three dimensions (eight intrusion items, eight avoidance items, and six hyperarousal items) described in the DSM-IV-TR to categorize PTSS [21]. It consisted of 22 statements that the responder rated on a 5-point scale (0 to 4) in terms of response to a specific stressful life event in the past week. Every PTSS score and the total score on the IES-R were measured. Cronbach’s coefficient alpha of IES-R in our study was 0.96. Cronbach’s coefficient alpha values of the three IES-R subscales of intrusion, avoidance, and hyperarousal, were 0.94, 0.76, and 0.87, respectively.

Regarding cutoffs for IES-R and CES-D scores, IES-R total scores above 25 represent high risk of PTSD [15], and CES-D scores above 16 represent high risk of clinical depression [18].

### Ethical considerations

This study was approved by the ethics committees of the Graduate School of Medicine of the University of Tokyo.

### Data analysis

Descriptive statistics were used for descriptive data. We used the Wilcoxon signed-rank test and the *t* test to compare variables related to psychopathology between the two time points and term of cancer diagnosis (original diagnosis/recurrence). Furthermore, we used two-way analysis of variance (ANOVA) to examine the main effect and interaction in terms of cancer diagnosis (original diagnosis/recurrence) and time point (T1/T2) on the IES-R score. Power analysis was conducted. Statistical significance was set at *p* < 0.05 [22]. All statistical analyses were done using SPSS 17.0 (SPSS, Chicago, IL).

## Results

**Fig. 1:**
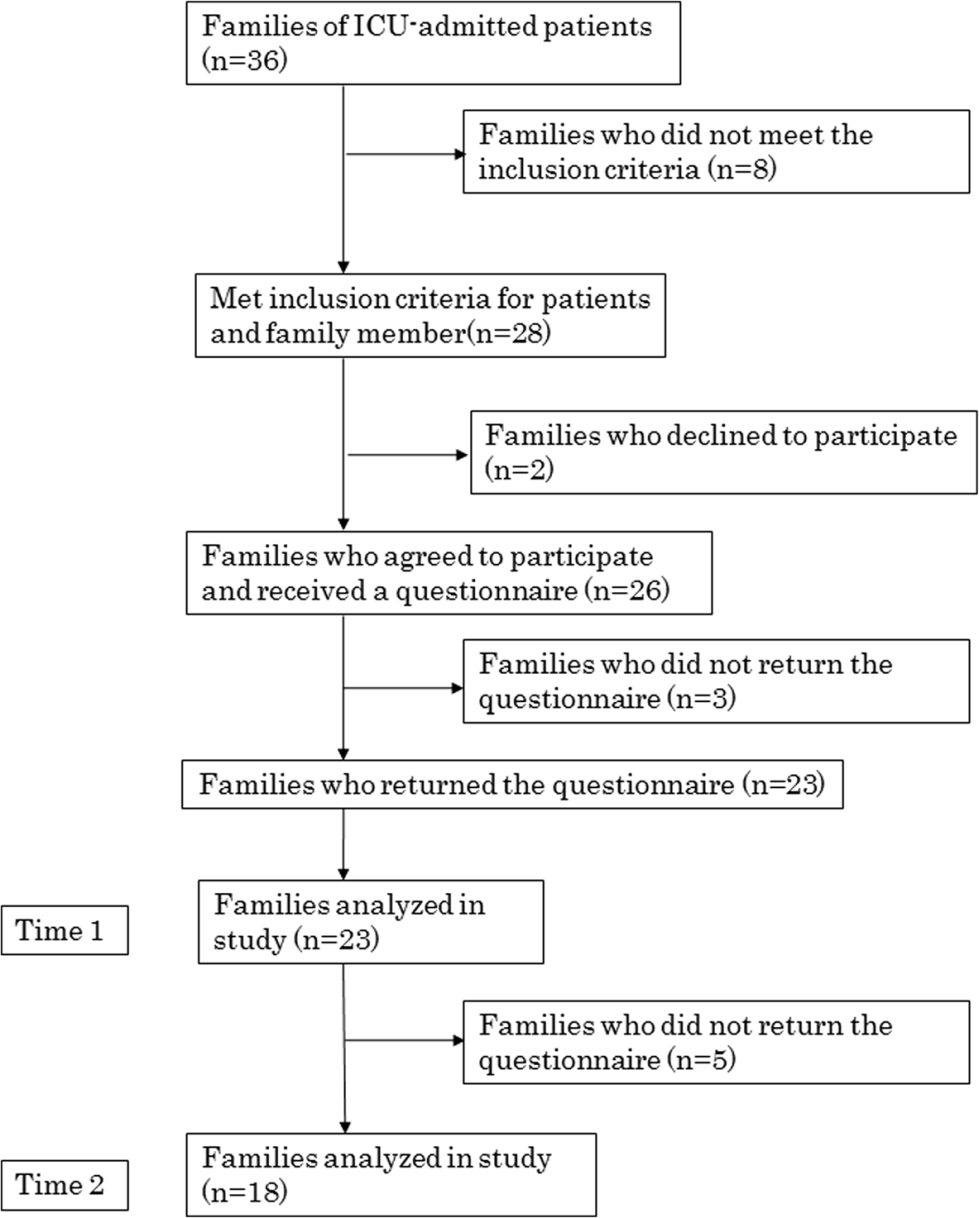
Enrollment of study participants. This chart begins with the 36 families of patients admitted to the ICU and illustrates the number of families who were excluded from the final analysis. The reasons eight families did not meet the inclusion criteria are categorized

Of the 26 families who agreed to participate and received questionnaires, 23 participants returned the questionnaires at T1. A total of 23 families were analyzed at T1, and 18 families were analyzed at T2 (Fig. 1).

Table 1 shows the demographics and clinical characteristics of patients and their accompanying family members and patients’ primary causative diseases for ICU admission.

**Table 1:**
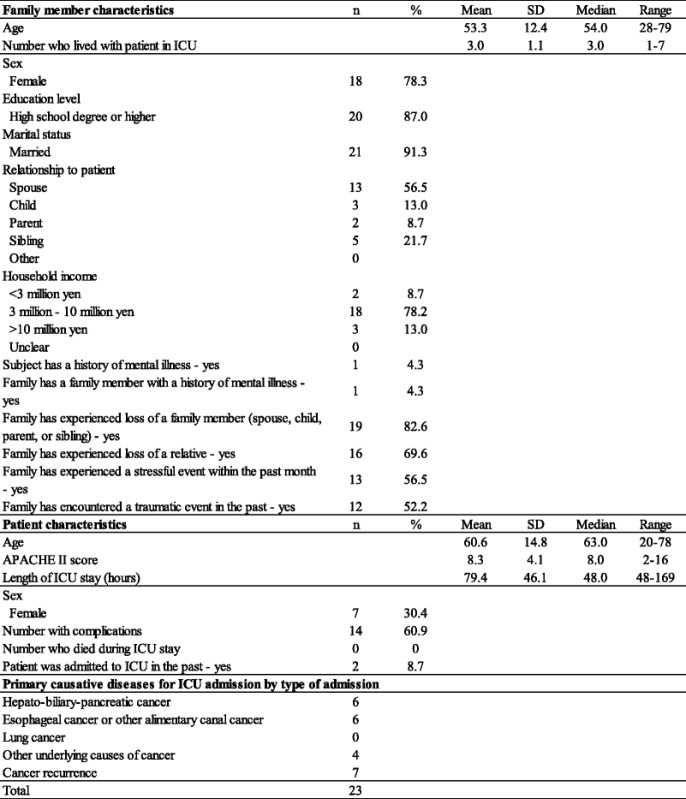
Characteristics of family members (*n* = 23) and patients admitted to the ICU (*n* = 23)

The measures of anxiety, depression, and symptom characteristics of PTSS by time point and cancer status are shown in Tables 2 and 3.

**Table 2:** Psychometric assessment of family members of ICU patients by time point

	Time point 1 (*n* = 23)			Time point 2 (*n* = 18)			*p*	*t* or *z*	*r* or *d*	post hoc
	Mean	SD	Median	Mean	SD	Median				
IES-R (total)	15.0	13.3	13.50	11.0	11.19	10.50	0.04^b^	−2.43	−0.57	0.73
Re-experience	6.79	7.17	4.0	4.42	5.06	3.4	0.06^b^	−1.98	−0.47	0.59
Arousal	4.83	5.70	32	2.13	3.18	1.8	0.38^b^	−0.44	−0.10	0.12
Avoidance	5.29	6.81	3.8	4.46	4.19	3.4	0.09^b^	−0.95	−0.22	0.22
CES-D	14.62	8.12	12.00	13.15	8.55	12.00	0.22^b^	−0.61	−0.14	0.14
STAI (state)	48.88	13.59	45.0	43.72	11.65	43.0	0.04^a^	2.41	0.51	0.53
STAI (trait)	45.11	5.3	38.0	–	–	–	–	–	–	–

*IES-R* Impact Event Scale-Revised, *CES-D* the Center for Epidemiological Studies Depression Scale, *STAI* the Spielberger’s State-Trait Anxiety Inventory

^a^Paired *t* test

^b^Wilcoxon signed-rank test

**Table 3:**
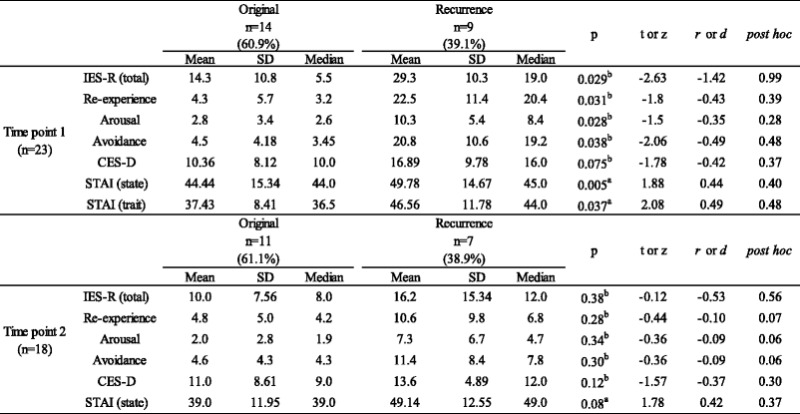
Psychometric assessment of family members of ICU patients by cancer status

*IES-R* Impact Event Scale-Revised, *CES-D* the Center for Epidemiological Studies Depression Scale, *STAI* the Spielberger’s State-Trait Anxiety Inventory

^a^Paired *t* test

^b^Wilcoxon signed-rank test

Mean total IES-R score and STAI-state score differed significantly from T1 to T2 (IES-R score *z* = −2.43, *p* = 0.04, *r* = −0.57, post hoc = 0.73; STAI-state *t* = 2.41, *p* = 0.04, *r* = 0.51, post hoc = 0.53). The percentage of family members whose IES-R scores were above the PTSD high-risk threshold of 25 was 21.7 % (5 of 23) at T1 and 11.1 % (2 of 18) at T2. The percentage of family members whose CES-D scores were above the clinical depression high-risk threshold of 16 was 21.2 % (7 of 23) at T1 and 16.7 % (3 of 18) at T2.

At T1, 14 of 23 (60.9 %) ICU admissions were for original cancer diagnoses and 9 of 23 (39.1 %) were for recurrent cancer diagnoses. At T2, 11 of 18 (61.1 %) ICU admissions were for original cancer diagnoses and 7 of 18 (38.9 %) were for recurrent cancer diagnoses.

Within 24 h of ICU admission, the mean IES-R score of families of patients with original and recurrent cancer admissions was 14.3 and 29.3, respectively. IES-R scores for families of patients with recurrent cancer diagnoses were significantly higher than scores for families of patients with original cancer diagnoses (*z* = -2.63, *p* = 0.029, *r* =-1.42, post hoc = 0.99). In terms of STAI-trait, a significant difference was observed between original and recurrent cancer diagnoses (*t* = 2.08, *p* = 0.037, *d* = 0.49, post hoc = 0.48).

At 3 months, the mean IES-R score of families of patients with original and recurrent cancer admissions was 10.0 and 16.2, respectively. There were no significant differences between these groups at 3 months (*z* = -0.12, *p* = 0.38, *r* = -0.53, post hoc = 0.56).

Two-way analysis of variance was used to assess the presence of differences in IES-R scores by time point (within 24 h/3 months later) and cancer status (original cancer/recurrent cancer). Time point was significantly associated with IES-R score (*F* = 1.751, *p* = 0.011, *df* = [1], *f* = 0.4, post hoc = 0.35). There was no significant main effect of cancer status or interaction effect of time point and cancer status (*F* = 1.751, *p* = 0.206, *df* = [1], *f* = 0.28, post hoc = 0.19).

## Discussion

Mean IES-R total scores in this study were as high as those seen in a previous study of families of patients with unplanned ICU admissions [23]. These family members felt severe psychiatric stress regarding expectations of death of the patient [24].

In addition, we revealed that the severity of PTSS of family members varied by the causative disease of the patient admitted to the ICU. Patients with unplanned ICU admissions had a higher disease severity than patients with recurrent cancer ICU admissions (APACHE II score of unplanned ICU admission was 20.4; APACHE II score of this study was 9.7) [23]. However, the PTSS of family members of patients with recurrent cancer ICU admissions was as severe as that of family members of patients with unplanned ICU admissions [23]. Thus, the psychiatric stress level of family members of patients with recurrent cancer ICU admissions was similar to that of family members of patients with unplanned ICU admissions despite the finding that the condition of patients with unplanned ICU admissions was more critical.

In early ICU admissions, this study also showed that family members of patients with recurrent cancer had more severe PTSS and anxiety than family members of patients with an original cancer diagnosis. A previous study reported that the low QOL of family members of recurrent cancer patients admitted to the general ward was related to the fact that the family members had believed the patient’s original cancer had been cured during the first admission [10]. Another study showed that families of patients with recurrent cancer felt fear when recalling the side effects the patient experienced during treatment of the original cancer [11]. These results indicated that family members of patients with cancer recurrence have a stronger psychiatric shock than family members of patients with original cancer. The results of this study were similar to these previous studies.

Recent studies have addressed the development of typical PTSD reactions and anxiety in relatives of ICU-treated adult patients. The current results showed that psychiatric shock and anxiety were reduced between early ICU admission and 3 months later. These results support the findings of Paparrigopoulos et al. (2006), who demonstrated that families of patients admitted to the ICU for various causes over a 6-month period have a high level of distress at ICU admission, but this distress level decreases 6 months later [25]. The Previous studies in Brazil and China showed that families of patients with recurrent cancer demonstrated development of typical PTSD reactions that were similar to those seen in families of patients with ICU admissions for various causes [6, 24]. In terms of depression, the results of this study differed from findings of Paparrigopoulos et al. [25].

Based on the cutoffs for IES-R and CES-D, even at T2, the percentages of families who were at high risk of PTSD and clinical depression were 11.1 % (2 of 18) and 16.7 % (3 of 18), respectively. These findings indicate that some families experience psychiatric burden for long periods, and psychiatric assessment and intervention are needed for families of patients admitted to the ICU due to exacerbation of cancer.

Families of patients with recurrent cancer were predisposed to anxiety, relative to families of patients with original cancer. A previous study reported that families of recurrent cancer patients have lower QOL than families of original cancer patients [10]. However, only few reports to date have compared the character traits of families of recurrent cancer patients and families of original cancer patients. The current study contributes to the scientific literature by offering insight into the relationship between cancer recurrence and development of psychiatric symptoms in family members of recurrent cancer patients.

### Limitations

This study has several limitations. First, this study was conducted at the medical/surgical ICU in a teaching and advanced treatment hospital. This could introduce selection bias regarding the state of the family. Second, this study was conducted by healthcare professionals providing high-quality medical care to severely ill patients and their families. This may have been a source of possible bias. Third, the questionnaire of this study was a Japanese self-administered questionnaire, which means that findings might be underestimated or overestimated. Fourth, the possibility of recall bias cannot be ruled out, especially as families were under a great deal of stress when completing the questionnaire. Fifth, the sample size of this study was small, and a larger sample size should be considered for future study designs. Sixth, this study was not able to evaluate PTSD because it did not involve any diagnostic interview. Finally, changes regarding PTSD diagnosis recently proposed in DSM-V were not taken into account [26]. The fact that the DSM-V expands the DSM-IV-TR’s three symptom clusters to four symptom clusters, it needs to be considered when interpreting the results of this study.

## Conclusions

Our results showed a difference in PTSS in family members of a cancer patient being admitted to the ICU with regard to cancer status from within 24 h to 3 months. However, some family members still have PTSS and depression 3 months later. It is necessary to protect families from mental distress after patients are discharged from the ICU. It is important that appropriate psychiatric support be provided to family members of these patients.

## Abbreviations

ANOVA, two-way analysis of variance; CAPS, the Clinical-Administered posttraumatic stress disorder scale; CES-D, the Center for Epidemiologic Studies Depression scale; IES-R, Impact of Event Scale-Revised; PTSD, Posttraumatic stress disorder; PTSS, Posttraumatic stress symptoms
